# Replacement of the Teeth

**Published:** 1853-10

**Authors:** 


*Replacement of the Teeth.*—Dr. Cushman, in the July No. of the American Journal of Dental Science, gives he details of a very successful case of replacement of a luxated tooth, the first bicuspis of the lower jaw, which had been extracted by mistake, and which was replaced, and in a few days grew tight, and since, some four years, has done very well, the tooth retaining its color and healthful appearance perfectly.

We have known of several cases, where teeth had been thus replaced, and subserved for years a very good purpose; one instance since we came to this city, a lad some 10 or 12 years of age, fell on the curb-stone, and knocked out a front upper incisor. My partner then, Dr. Rostaing replaced it, and secured it by ligature to the contiguous teeth. It soon grew fast, and for years, and we know not but as yet, subserves a good purpose. We recollect one case, an inferior molar, which was drawn for the tooth-ache, and was replaced, and lasted after this, for many years.; we think not less than eight or ten The single fanged teeth, are however, the ones best adapted. for this operation, and such, would more generally do well.

We should always replace a front tooth, if sound, and displaced by mistake or accident, if the alveolus was not materially injured.

